# Ruminant and human brucellosis situation in Türkiye and the Caucasus

**DOI:** 10.1007/s11250-025-04525-1

**Published:** 2025-07-11

**Authors:** Ipek Keskin Fernandez-Georges, Sheina Macy Manalo, Margarida Arede, Giovanna Ciaravino, Daniel Beltrán-Alcrudo, Jordi Casal, Eran Raizman, Jeyhun Aliyev, Tengiz Chaligava, Tigran Markosyan, Alberto Allepuz

**Affiliations:** 1 Department of Animal Health and Anatomy, Faculty of Veterinary Medicine, Autonomous University of Barcelona (UAB), Barcelona, Spain; 2 Department of Veterinary Paraclinical Sciences, College of Veterinary Medicine, University of the Philippines Los Baños, Los Baños, Philippines; 3 Food and Agriculture Organization of the United Nations (FAO), Regional Office for Europe and Central Asia, Budapest, Hungary; 4 Azerbaijan State Agricultural University, Ganja, Azerbaijan; 5 Veterinary Department of National Food Agency, Tbilisi, Georgia; 6 Scientific Center for Risk Assessment and Analysis in Food Safety Area, Closed Joint-Stock Company, Yerevan, Armenia

**Keywords:** Brucellosis, Epidemiology, Caucasus, Türkiye, Surveillance, Public health

## Abstract

Brucellosis, a disease caused by *Brucella* spp., is one of the most prevalent zoonoses in the world and remains endemic in the Mediterranean Basin, the Near East, South America, and parts of Africa. Given the negative impact it has on public and animal health, food safety and livelihoods, sustained campaigns for its control and eradication are crucial. This study aimed to provide a better understanding of brucellosis status in domestic ruminants in Armenia, Azerbaijan, Georgia and Türkiye by looking into the spatial and temporal distribution of the disease in terms of the standardized morbidity ratio/standardized incidence ratio (SMR/SIR), as well as the distribution in humans (SIR). The results of this study highlight areas of higher risk of disease in ruminants, especially near border areas between endemic countries, and the uneven distribution of human cases. Factors that hamper the control of brucellosis in Türkiye and the Caucasus include a lack of sustained funding and adequate animal identification and traceability systems (NAITSs) to identify animals for disease management interventions (i.e., vaccination, test-and-slaughter) and allow for the effective control of animal movement and trade within the countries.

**Supplementary Information:**

The online version contains supplementary material available at 10.1007/s11250-025-04525-1.

## Introduction

Brucellosis is an animal and human infection caused by the genus *Brucella* spp, within the family *Brucellaceae*, of the order Rhizobiale in the class Alphaproteobacteria. Cattle are primarily infected by *B. abortus*, but can also be infected by *B. melitensis* and occasionally with *B. suis*. The main cause of infection in sheep and goats is *B. melitensis*. The species which are most pathogenic and transmissible to humans are *B. melitensis, B. abortus,* and *B. suis*. Although it is not the subject of this study, *B. suis* is prevalent in many countries where pigs are raised. Infection with Brucella in animals can cause abortion, infertility, retained placenta, orchitis, epididymitis and, in rare cases, arthritis. The pathogen is shed in uterine discharge and body fluids such as milk, urine and semen (World Organisation for Animal Health 2022a). The disease usually spreads within a herd through ingestion and direct contact with contaminated materials, such as the aborted foetuses and genital discharges of infected animals, and feeding of contaminated colostrum and milk. Other routes of infection include exposure via the respiratory tract, conjunctiva and damaged skin or mucous membranes, and iatrogenic infection from contaminated syringes (Corbel 2006; Spickler 2018).

In humans, brucellosis is considered a foodborne and occupational disease (Havas 2011; Yumuk and O’Callaghan 2012). Transmission from animals to humans occurs via three main routes: consumption of contaminated and untreated raw milk and milk products, direct or indirect contact with infected animals and their tissues, and inhalation of infected aerosols (Can 2010; Aypak et al. 2012; Yumuk and O’Callaghan 2012; Kracalik et al. 2016; Mengele et al. 2023). Farming professionals (i.e., farmers, slaughterhouse workers, shepherds, sheep shearers, butchers, veterinarians, artificial insemination technicians, and anyone else working along the value chain of brucellosis-susceptible animals and their products) and laboratory personnel are at higher risk of infection in endemic areas (Can 2010; Yumuk and O’Callaghan 2012; Mengele et al. 2023). In addition to acute febrile illness, Brucellosis can have serious consequences for public health by affecting the musculoskeletal, cardiovascular and central nervous systems (World Organisation for Animal Health 2022a). The World Health Organization recommends the standard human brucellosis case definition be “acute or insidious onset, with continued, intermittent or irregular fever of variable duration, profuse sweating, fatigue, anorexia, weight loss, headache, arthralgia, and generalized aching” and confirmed with laboratory diagnosis (Corbel 2006). Although human brucellosis has long been recognized as a major cause of morbidity worldwide, the annual prevalence of the disease is unclear due to underdiagnosis and underreporting of human and ruminant cases in endemic areas (Holt et al. 2011; Havas et al. 2012; Franc et al. 2018; Avila-Granados et al. 2019; Laine et al. 2022; Moreno et al. 2022; Mengele et al. 2023). The actual incidence in humans each year is estimated to be between five and 12.5 million cases worldwide (Mengele et al. 2023).

Given the negative impact of brucellosis on public and animal health, food safety and livelihoods, sustained campaigns to control and eradicate it in livestock are crucial. It remains endemic in countries of the Mediterranean Basin, the Near East, South America, and parts of Africa (Hull and Schumaker 2018). In these regions, factors such as low vaccination coverage, reinfection of herds, ineffective disease surveillance, reluctance of farmers to remove seropositive animals from herds due to lack of compensation from regulatory authorities, and insufficient funding to sustain long-term surveillance and control activities, compromise disease control (Khoshnood et al. 2022).

Ruminant production is especially important in Türkiye and the Caucasus, contributing significantly to employment, rural livelihoods, and food security (Rukhkyan 2003; Food and Agriculture Organization 2004, 2012; Punjabi 2020). In 2020, the Gross Production Value (GPV) for cattle, calculated by GPV for cattle divided by the GPV for all domestic species, was highest in Armenia (66.4%) and Türkiye (65.0%) compared to 57.7% in Georgia and 56.4% in Azerbaijan (Food and Agriculture Organization 2022a). This paper only discusses surveillance and vaccination, although other control measures for brucellosis are implemented in these countries. In brief, active surveillance is mostly carried out in Armenia and Azerbaijan. In Armenia, passive surveillance is voluntary since farmers and veterinarians are not required to record abortions. In Georgia, passive surveillance has been implemented since 2018. In Türkiye, both active and passive surveillance are carried out. In terms of brucellosis vaccination by country at the time of this study: Armenia was not vaccinating; Azerbaijan was vaccinating all female cattle with S-19 and small ruminants (SR) aged 3–8 months with Rev-1 and all non-pregnant females; Georgia was vaccinating female calves with S-19/RB-51 and replacement lambs aged 3–12 months with Rev-1; and Türkiye was vaccinating female calves, lambs and kids aged 3–6 months and breeding males with S-19 and Rev-1 conjunctival vaccine, respectively, and re-vaccinating female calves aged 4–12 months after the first vaccination.

The main objectives of this study were to describe and interpret the temporal and spatial trends of ruminant brucellosis (based on serosurveillance programs) in Armenia, Azerbaijan, Georgia, and Türkiye, to identify the risk areas for humans and livestock, and to evaluate the progress of surveillance and control activities.

## Materials and methods

### Data collection and management

Information on national brucellosis surveillance programs and vaccination data for cattle and SR, as well as ruminant demographics, were obtained from experts involved in this study from Armenia, Azerbaijan, Georgia, and Türkiye, as part of a project on ruminant diseases in the Black Sea Basin (Arede et al. 2023). Based on the surveillance data available, different time frames were established for each country: Armenia (2014–2020), Azerbaijan (2016–2020), Georgia (2014–2017), and Türkiye (2017–2020).

Under the Armenian surveillance program, blood samples were taken twice a year (in spring and autumn) from all adult cattle, and once a year (in spring) from all adult female SR (Government of the Republic of Armenia 2022).

Under the Azerbaijan surveillance program, blood samples were taken from cattle and SR of all ages that had not been vaccinated against brucellosis. The national animal identification and traceability system (NAITS) was not in place at the time of sampling, so small incisions were made in the ears of vaccinated ruminants to enable identification of these animals in subsequent surveys (Ministry of Agriculture of the Republic of Azerbaijan 2020; Khatibi et al. 2021).

In Georgia, blood samples were taken from ear-tagged female large ruminants up to 6 months of age and only animals that tested negative for brucellosis were vaccinated after testing. If the sampled animals had not been previously identified, they were ear-tagged at the time of sampling to ensure traceability (Government of Georgia 2015).

In Türkiye, under the passive surveillance program, following the reporting of abortions suspected of being caused by brucellosis, samples were sent to the laboratory (Circular on Combating Animal Diseases and Animal Movement Control 2023).

Data on the number of human cases at the first administrative level were obtained from the Ministry of Health in Armenia, the Electronic Integrated Disease Surveillance System in Azerbaijan, and the Communicable Disease Department at the National Center for Disease Control and Public Health (NCDC) in Georgia. The incidence of brucellosis at the provincial level in Türkiye was obtained from the website of the General Directorate of Public Health (Ministry of Health of the Republic of Türkiye 2017). Human population data were obtained from the websites of the Statistical Committees of Armenia, Azerbaijan and Georgia (Statistical Committee of the Republic of Armenia 2023; State Statistical Committee of the Republic of Azerbaijan 2023; National Statistics Office of Georgia 2023). The time frame for human brucellosis cases in Armenia, Azerbaijan, and Georgia was set from 2016 to 2020, while for Türkiye only the year 2017 was used, as it was the only time frame with data available online.

### Descriptive and statistical analysis

For Türkiye, only passive surveillance data reporting new infected herds were available, while for the other countries, only active surveillance data on the number of positive and tested animals were available. The cumulative incidence (i.e., new cases divided by the total population) per year was computed only for Türkiye at herd level, while the yearly prevalence at animal level (i.e., positive animals divided by tested animals) was calculated for Armenia, Azerbaijan, and Georgia at country level. The 95% confidence intervals of incidence and prevalence were calculated on the VassarStats statistical computation website (http://vassarstats.net/). Armenia, Azerbaijan, and Türkiye reported data on SR and cattle separately, so we used the Pearson’s chi-square test and calculated the relative risk (RR) with its 95% confidence interval to estimate whether there were differences between groups in the prevalence or incidence. EpiInfo 7.2.5.0. was used for these calculations (https://www.cdc.gov/epiinfo/). To examine the relationship between positive tested animals and human brucellosis cases, Spearman correlation analysis was performed using RStudio version 4.4.0.

### Spatial distribution

#### Brucellosis in ruminants

To represent the spatial distribution of the risk of ruminant brucellosis in these countries we used the standardized morbidity ratio/standardized incidence ratio (SMR/SIR), which is commonly used to represent the spatial pattern of disease distribution (Pfeiffer et al. 2008). This ratio is calculated as the number of seropositive ruminants divided by the number of expected seropositive ruminants in a specific area and therefore represents the excess of risk (if any) in that area. For Armenia, Azerbaijan and Georgia, the SMR for all ruminants was obtained by using the following formula:

$${SMR}_{i}=\frac{{C}_{i,t}}{{E}_{i,t}}$$

where *C* denotes the number of seropositive ruminants, *E* is the number of expected seropositive ruminants, *i* is the administrative division, and *t* is the year.

Except for Georgia, where data for SR and cattle were not provided separately, the number of expected seropositive ruminants (*E*) was computed as the sum of the brucellosis prevalence in SR (*SRPrev*) times the number of SR tested (*SRTest*) plus the prevalence in large ruminants (*LRPrev*) times the number of large ruminants tested (*LRTest*):

$${E}_{i,t}=\left(SRP{rev}_{j,t}\right)\times \left({SRTest}_{i,t}\right)+ \left(LRP{rev}_{j,t}\right)\times \left({LRTest}_{i,t}\right)$$

where *j* is the country, *t* is the year, and *i* is the administrative level.

In Türkiye, the SIR was computed with the same formula as the SMR, but with new herds infected by administrative unit as the numerator.

$${SIR}_{i}=\frac{{O}_{i,t}}{{E}_{i,t}}$$

For Türkiye, the expected number of herds in which ruminants are aborted was computed as the sum of SR herd incidence in the country multiplied by the SR herd numbers in each administrative division plus large ruminant herd incidence in the country multiplied by the large ruminant herd numbers per administrative division and year.

#### Human brucellosis

The spatial distribution of human brucellosis risk in Armenia, Azerbaijan, and Georgia was calculated using the same SIR formula as for ruminant brucellosis. In this calculation, the expected cases were calculated as the human cases in the respective countries multiplied by the human population in each administrative division of these countries per year.

For Türkiye, the human brucellosis incidence per province level for 2017 was used.

The obtained ratios of animal and human brucellosis were both mapped using QGIS (version 3.16.11) within the corresponding time periods.

#### Vaccination coverage

At the time of this study, brucellosis vaccination was not performed in Armenia. Brucellosis vaccination coverage per year was calculated separately for cattle and SR, using different time periods for Azerbaijan (2017–2021), Georgia (2016–2022), and Türkiye (2017–2020). The numerators in these calculations were the number of cattle and SR vaccinated per year but different methods were used to calculate the denominators. Turkish population data that could be used as a denominator were available, however exact population data for Azerbaijan and Georgia were not. Therefore, the estimated proportion of the expected total population for vaccination against brucellosis, decided upon by the national veterinary authorities, was used to calculate denominators for these countries. This data comprised females and males under six months of age and between 12 and 24 months old, and males over two years old for SR, as well females under two years old for cattle.

## Results

### Descriptive analysis

#### Brucellosis in ruminants

Brucellosis prevalence for Armenia, Azerbaijan, and Georgia is presented in Table 1. In Armenia and Azerbaijan, the prevalence was found to increase over time, especially in SR in Armenia (from 0.15% in 2014 to 0.68% in 2019) and in cattle in Azerbaijan (from 0.11% in 2016 to 2.17% in 2020). In both countries, the prevalence in SR was higher than in cattle in most years, except in Azerbaijan in 2020, when the prevalence was nearly two times higher in cattle. In Georgia, prevalence remained more or less constant. Table 2 shows the incidence of brucellosis determined by passive surveillance in SR and cattle in Türkiye. The herd incidence ranged from 0.01% and 0.06% between 2017 and 2020 and was 2.0–3.6 times higher in cattle than in SR.

**Table 1:** Brucellosis prevalence in small ruminants and cattle in Armenia, Azerbaijan, and Georgia, based on active surveillance data (2014–2020). SR: small ruminants, ns: non-significant, RR: risk ratio, CI: confidence interval

Year	Species	Number of positive animals	Animals tested	Prevalence (%)	95% CI	*p*-value	RR	95% CI
Armenia								
2014	SR	518	356,838	0.15	0.13–0.16	< 0.05	0.89	0.80–0.99
	Cattle	1,017	626,427	0.16	0.15–0.17			
2015	SR	609	370,510	0.16	0.15–0.17	ns	1.00	0.90–1.10
	Cattle	1,048	636,022	0.16	0.15–0.17			
2016	SR	738	346,758	0.21	0.20–0.23	ns	1.03	0.94–1.13
	Cattle	1,221	590,722	0.21	0.20–0.22			
2017	SR	914	314,256	0.29	0.27–0.31	< 0.05	1.14	1.05–1.24
	Cattle	1,353	532,160	0.25	0.24–0.27			
2018	SR	995	305,949	0.33	0.31–0.35	< 0.05	1.13	1.04–1.22
	Cattle	1,458	506,805	0.29	0.27–0.30			
2019	SR	2,151	316,267	0.68	0.65–0.71	< 0.05	1.11	1.05–1.17
	Cattle	3,142	511,630	0.61	0.59–0.64			
2020	SR	1,816	301,244	0.60	0.58–0.63	< 0.05	1.67	1.57–1.78
	Cattle	1,952	542,860	0.36	0.34–0.38			
Azerbaijan								
2016	SR	651	156,837	0.42	0.38–0.45	< 0.05	3.77	3.41–4.16
	Cattle	943	858,611	0.11	0.10–0.12			
2017	SR	465	159,087	0.29	0.27–0.32	< 0.05	2.34	2.10–2.60
	Cattle	1,117	894,495	0.12	0.11–0.13			
2018	SR	468	155,990	0.30	0.27–0.33	< 0.05	1.85	1.65–2.07
	Cattle	913	563,367	0.16	0.15–0.17			
2019	SR	682	19,319	3.53	3.28–3.80	< 0.05	4.62	4.18–5.11
	Cattle	836	112,486	0.74	0.69–0.80			
2020	SR	1,203	122,777	0.98	0.93–1.04	< 0.05	0.46	0.41–0.51
	Cattle	432	19,942	2.17	1.97–2.38			
Georgia								
2014	Ruminants	2,506	126,515	1.98	1.90–2.06	-	-	-
2015	Ruminants	1,013	65,626	1.54	1.45–1.64	-	-	-
2016	Ruminants	4,339	193,517	2.24	2.18–2.31	-	-	-
2017	Ruminants	2,341	153,063	1.53	1.47–1.59	-	-	-

**Table 2:** Brucellosis incidence in small ruminants and cattle in Türkiye, based on passive surveillance data (2017–2020). SR: small ruminants, RR: risk ratio, CI: confidence interval

Year	Species	New outbreaks	Number of herds	Incidence (%)	95% CI	P-value	RR	95% CI
Türkiye								
2017	Cattle	778	1,452,874	0.05	0.05–0.06	< 0.05	1.90	1.58–2.29
	SR	128	453,963	0.03	0.02–0.03			
2018	Cattle	2,078	1,400,123	0.15	0.14–0.15	< 0.05	3.48	2.98–4.05
	SR	179	419,230	0.04	0.04–0.05			
2019	Cattle	2,009	1,423,102	0.14	0.14–0.15	< 0.05	3.66	3.13–4.27
	SR	174	451,020	0.04	0.03–0.04			
2020	Cattle	857	1,439,294	0.06	0.06–0.06	< 0.05	2.00	1.66–2.40
	SR	129	433,353	0.03	0.03–0.04			

#### Human brucellosis

The incidence of human brucellosis as cases per 100,000 population varied across regions in Armenia, Azerbaijan and Georgia. The details of brucellosis incidence calculated in cases per 100,000 population in Armenia, Azerbaijan, and Georgia between 2016 and 2020 and the incidence per province in Türkiye in 2017 are presented in Tables 1, 2, and 3 in the supplementary material.

**Table 3:** Correlation between human brucellosis cases and positive tested cattle and small ruminants in Armenia, Azerbaijan (2016–2020) and Türkiye (2017). SR: small ruminants

Armenia			Human	Cattle	SR
Spearman’s rho	Correlation Coefficient	Human	1	0.3077062	0.4238831
		Cattle	0.3077062	1	0.6217568
		SR	0.4238831	0.6217568	1
Azerbaijan			Human	Cattle	SR
Spearman’s rho	Correlation Coefficient	Human	1	0.2996569	0.2495225
		Cattle	0.2996569	1	0.5457336
		SR	0.2495225	0.5457336	1
Türkiye			Human	Cattle	SR
Spearman’s rho	Correlation Coefficient	Human	1	0.1201768	0.1008998
		Cattle	0.1201768	1	0.4995900
		SR	0.1008998	0.4995900	1

Armenia had the highest and lowest incidence of human brucellosis at country level in 2017 (191.5) and 2020 (74.9), respectively. Incidence values varied between regions and similarly, within regions tended to increase and/or decrease in successive years. The passive nature of data collection and low public awareness may have influenced these fluctuations. In Azerbaijan, the highest incidence at country level was observed in 2018 (429.2) and the lowest in 2020 (115.2). In Georgia, as observed in animals, incidences observed in humans varied by year and region. This can depend on insufficient population awareness and on the fact that surveillance was passive. The highest incidence at country level was observed in 2017 (72.4). Similar to Armenia and Azerbaijan, the lowest incidence was observed in 2020 (41.4). In Türkiye, the incidence rates ranged from 0.4 to 82.4 in 2017.

### Spatial distribution

#### Brucellosis in ruminants

**Fig. 1:**
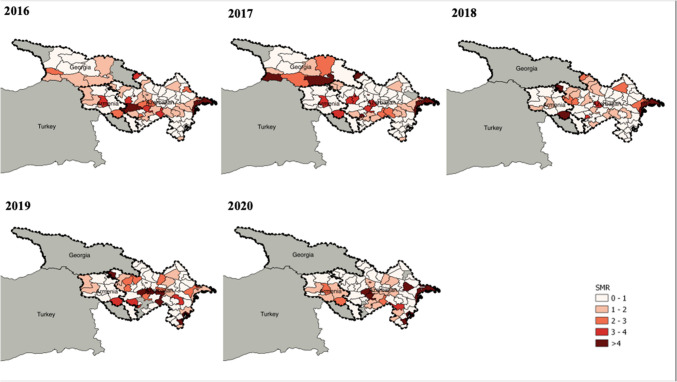
Standardized morbidity ratio (SMR) of brucellosis in ruminants in Armenia, Azerbaijan and Georgia (2016–2020)

**Fig. 2:**
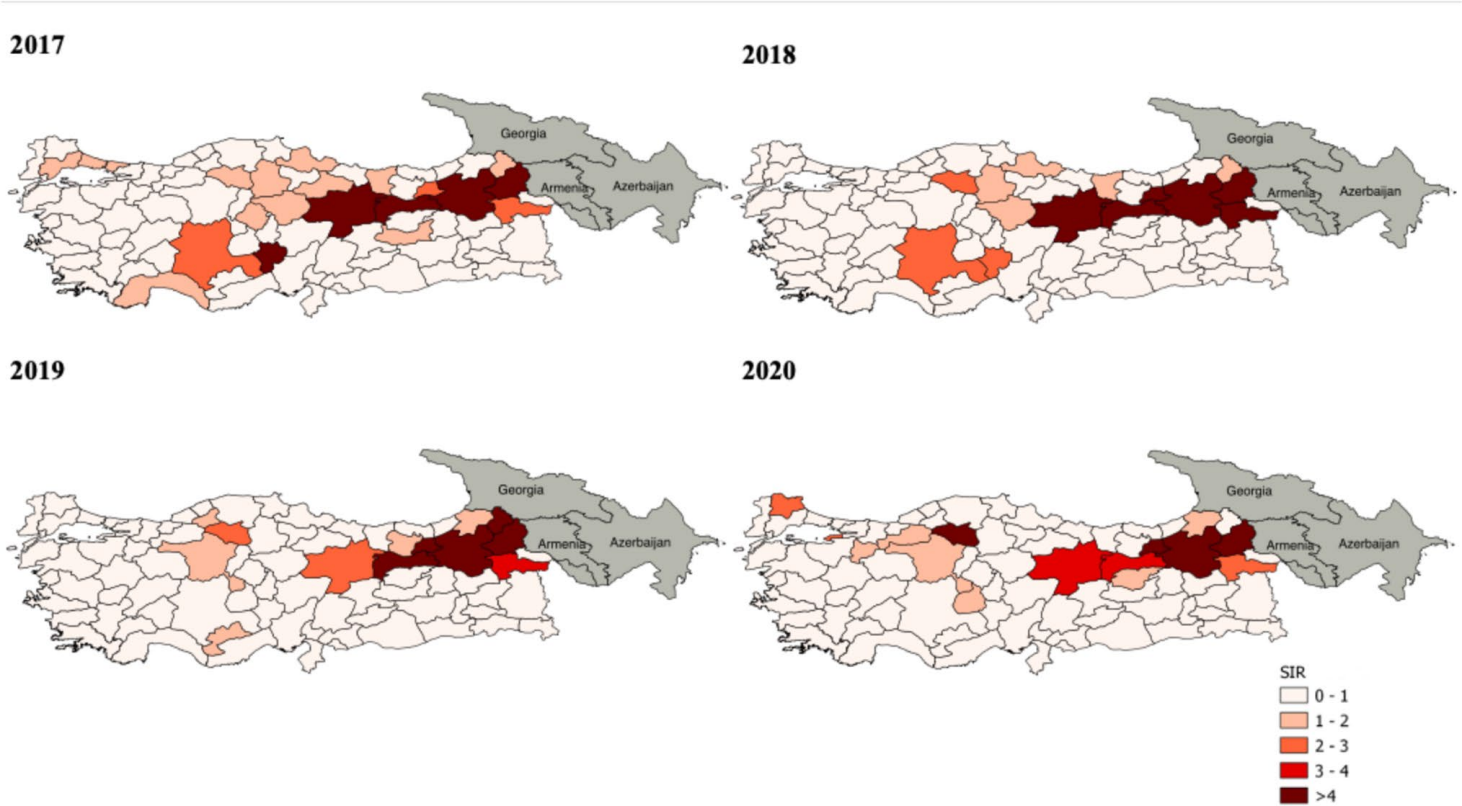
Standardized incidence ratio (SIR) of brucellosis in ruminants in Türkiye (2017–2020)

The SMR values of brucellosis in ruminants in Armenia, Azerbaijan, and Georgia between 2016 and 2020 are shown in Fig. 1, and the SIR in Türkiye in Fig. 2. The highest risk in Armenia was found in the center of the country (Kotayk marz) and for Azerbaijan, in the East and center of the country. In Georgia, the SMR was highest in the South (bordering Armenia and Azerbaijan), and West (bordering Türkiye). In eastern and western Georgia, active surveillance focused on large ruminants which increased the number of positive animals tested, having an impact on the prevalence. In Türkiye, the incidence was highest in the eastern and central provinces.

#### Human brucellosis

**Fig. 3:**
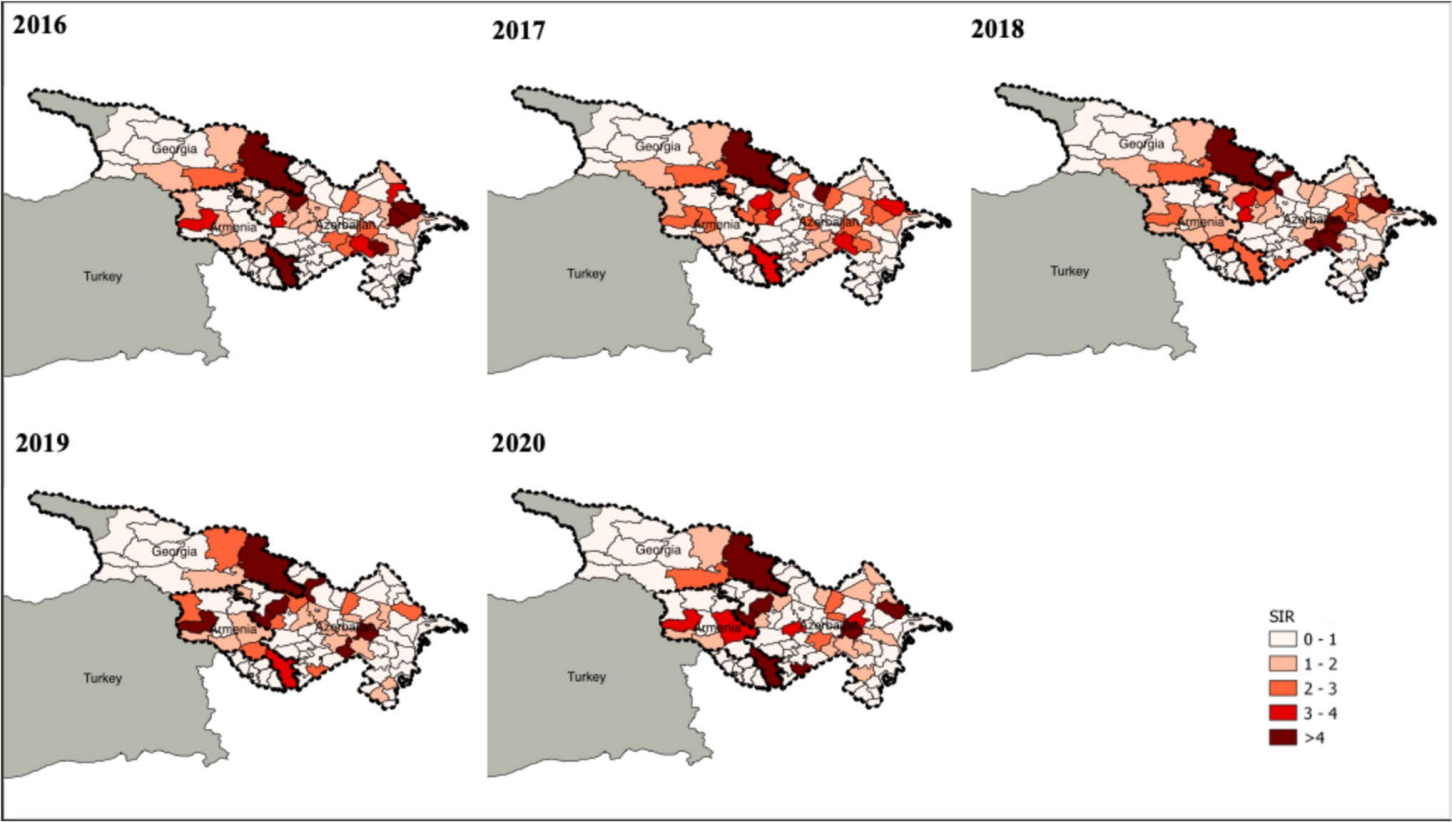
Standardized incidence ratio (SIR) of human brucellosis in Armenia, Azerbaijan, and Georgia (2016–2020)

**Fig. 4:**
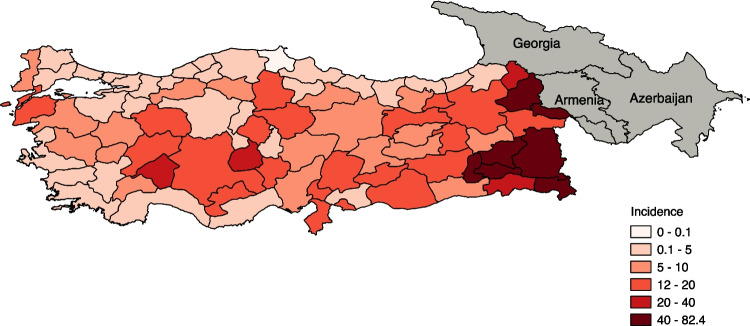
Human brucellosis incidence in Türkiye (2017)

Figure 3 shows the SIR of human brucellosis in Armenia, Azerbaijan and Georgia between 2016 and 2020. Figure 4 shows the human brucellosis incidence in Türkiye in 2017. The human brucellosis SIR did not change significantly between 2016 and 2020 in Armenia, Georgia and Azerbaijan, except for a slight increase in Armenia in 2019, and in Azerbaijan in 2016 and 2018. Similar to ruminant brucellosis patterns, the highest human risk in Georgia was observed in the southern, southeastern and southwestern regions bordering Armenia, Azerbaijan, and Türkiye. The regions of Armenia that border the Islamic Republic of Iran and Azerbaijan held the greatest risk, followed by those bordering Türkiye. In Azerbaijan, the highest risk was in the East, the center, and in districts bordering Armenia. In Türkiye, the eastern, southeastern, and central provinces had the highest risk in 2017, but the disease was present in 80 out of 81 provinces.

In the current study, the analysis of the Spearman rank correlation coefficient (rho) showed a poor correlation between positive tested cattle and SR and human brucellosis cases, with the value ranging between 0.100 and 0.423 for Armenia, Azerbaijan and Türkiye. This suggests that human brucellosis cases are weakly related to positive tested animals in these countries. The results are shown in Table 3.

In Georgia, similar to Armenia, Azerbaijan and Türkiye, the correlation between human cases of brucellosis and positive ruminant tests was weak, with a value of 0.104 (Table 4).

**Table 4:** Correlation between human brucellosis cases and positive tested ruminants in Georgia (2016–2020)

Georgia			Ruminants	Human
Spearman’s rho	Correlation Coefficient	Ruminants	1	0.1043751
		Human	0.1043751	1

### Vaccination coverage

The average vaccination coverage calculated for Azerbaijan, Georgia, and Türkiye was not high enough to control brucellosis effectively. The details of brucellosis vaccination coverage in Azerbaijan, Georgia, and Türkiye for different time periods is shown in Table 5, with low coverage rates in bold.

**Table 5:** Brucellosis vaccination coverage in small ruminants and cattle in Azerbaijan, Georgia, and Türkiye (2016–2022). M: millions

Year	Small ruminants			Cattle		
	Vaccinated population	Planned vaccination	Vaccination coverage (%)	Vaccinated population	Planned vaccination	Vaccination coverage (%)
Azerbaijan						
2017	1,754 M	1,5 M	116.9	0,3983 M	1,94 M	**21.0**
2018	1,4777 M	1,55 M	95.3	0,3961 M	0,4 M	99.0
2019	1,341 M	5,516 M	**24.3**	0,3915 M	1,2846 M	**30.5**
2020	4,81 M	5,52 M	87.1	1,1404 M	1,285 M	88.8
2021	1,38 M	2 M	**69.0**	0,3671 M	1 M	**36.7**
Average			78.5			55.0
Georgia						
2016	-	-	-	0,03584 M	0,24068 M	14.9
2017	-	-	-	0,23275 M	0,22743 M	102.3
2018	-	-	-	0,1545 M	0,21973 M	**70.3**
2019	0,25026 M	0,21048 M	118.9	0,20883 M	0,21738 M	96.0
2020	0,08367 M	0,22405 M	**37.3**	0,12292 M	0,23145 M	**53.1**
2021	0,011514 M	0,22608 M	**5.09**	0,18565 M	0,23215 M	80.0
2022	0,08777 M	0,21595 M	**40.2**	0,14578 M	0,21343 M	**68.3**
Average			50.5			69.3
Year	Vaccinated population	Target Population	Vaccination coverage (%)	Vaccinated population	Target Population	Vaccination coverage (%)
Türkiye						
2017	4,887 M	12,542 M	**39.0**	2,2885 M	4,3910 M	**52.1**
2018	5,468 M	11,984 M	**45.6**	2,608 M	4,6199 M	**56.4**
2019	5,127 M	12,439 M	**41.2**	2,731 M	4,8180 M	**56.7**
2020	4,555 M	15,115 M	**30.1**	3,143 M	4,8224 M	**65.2**
Average			39.0			57.6

## Discussion

The surveillance prevalence for ruminants in Armenia, Azerbaijan, Georgia, and Türkiye obtained in this study was lower than in previous studies (Mamisashvili et al. 2013; Kara et al. 2014; Baghiyan and Markosyan 2015; Khatibi et al. 2021), which may indicate an improvement in the effectiveness of the control programs or different degrees of risk among areas. Nevertheless, the eradication of ruminant brucellosis in countries examined in the current study remains challenging due to several factors. One of these factors is the absence of fully functional NAITSs, which is at the core of improving the effectiveness of activities such as surveillance, vaccination planning, tracing, outbreak response, and disease notification (Zhang et al. 2018; World Organisation for Animal Health 2022b). NAITS are at different stages of implementation in the region. Armenia began pilot implementation for cattle in early 2022, Azerbaijan began phased implementation for cattle in 2022, and Georgia began nationwide use of the system in early 2022 for individual cattle and SR at herd level (Austrian Development Agency 2018; Aliyev 2021; Center for Agribusiness and Rural Development 2018; Food and Agriculture Organization 2022b). In contrast, Türkiye has a national system (TURKVET) for identifying and registering cattle and SR that was implemented for relevant animal species starting in 2001 and 2010, respectively (Circular on Combating Animal Diseases and Animal Movement Control 2023; Directorate General of Food and Control, Ministry of Agriculture and Forestry Report 2024). The accurate census of ruminants is another factor that can be challenging in Armenia, Azerbaijan and Georgia. In 2015, Baghiyan and Markosyan found that the livestock population data provided by the State Agency of Statistics in Armenia were incorrect, and thus, the planning of government test-and-slaughter programs did not always cover all the animals which were at risk. In Azerbaijan, the data obtained from regional veterinarians are used for program planning (Khatibi et al. 2021). Similarly to Armenia, Georgia uses livestock data from the National Statistics Office of Georgia (National Statistics Office of Georgia 2023).

In Armenia, about 80% of rural households are engaged in livestock production, mainly pasture-based cattle farming. A third of households are involved in small ruminant production, which is unevenly distributed across the country and based on the availability of pastures (Rukhkyan 2003; Ministry of Agriculture of the Republic of Armenia 2010). The majority of livestock in Azerbaijan is reared on small farms with a variety of animal species, with ruminants being the most common. Goats account for 1–2% of the animals kept by the majority of small ruminant farmers. Common mountain pastures may be the summer grazing area for village-based livestock, which graze on common ground in autumn and winter (Khatibi et al. 2021). In Georgia, sheep farming largely relies on traditional pastoral practices, and mixed livestock populations from multiple owners are grazed on unfenced village pastures and mountain pastures in adjacent areas. In Georgia, pastures contaminated with *Brucella* spp., through discharge from parturition during winter, can cause infections in grazing animals (Havas et al. 2012). In Türkiye, the intensive family-run cattle farms that existed until the 1980 s were first replaced by commercial farms and then by modern, government-supported farms. Even today, sheep farming in the country is generally a family business and 90% of breeding is based on pasture, unless pastures are covered in snow or it rains heavily (Directorate General of Agricultural Enterprises 2020; Çiçek et al. 2022; Directorate General of Livestock 2024). Cattle movement between herds is positively associated with brucellosis (Cowie et al. 2014). In the border regions of Georgia and Türkiye animals share common grazing pastures. Poor control of livestock movements in seasonal pastures and of transboundary animal movements in these countries are risk factors which facilitate the introduction and transboundary spread of *Brucella* spp. with different incidences over time.

Previous studies have found that brucellosis prevalence in Türkiye varied depending on the geographical regions (Şahin et al. 2008). The current study also revealed that the brucellosis risk in ruminants is not uniformly distributed in Türkiye, with the highest risk observed in some provinces of the Eastern Anatolia region, bordering Armenia, Georgia, and the Islamic Republic of Iran. Some provinces in Central Anatolia also presented a high risk. These findings are consistent with those of Demeli and Findik (2021), where cattle and SR brucellosis outbreaks were more frequent in the Eastern and Central Anatolia regions.

Our study found a lower proportion of cattle infected with *Brucella* spp. than SR in Armenia and Azerbaijan, in contrast with the passive surveillance results from Türkiye, where the incidence in cattle was higher than in SR. This could be explained by the fact that passive surveillance relies upon voluntary reporting. In passive surveillance for brucellosis, a higher incidence is typically reported in cattle than in small ruminants, which is largely due to cattle receiving more veterinary attention, particularly in the dairy sector, where reproductive issues are closely monitored and more likely to be reported. In contrast, brucellosis in sheep and goats often goes unnoticed, especially in extensive farming systems where abortions may not be observed or reported. The economic value of cattle and stronger surveillance infrastructure further contribute to this reporting bias. As a result, passive surveillance tends to underrepresent the true burden of disease in small ruminants. Therefore, the results of this study may be attributed to factors such as the herd size of cattle and SR, as smaller herds provide less chances for contact between animals, as well as farm type, vaccination coverage, or local herd management system, among others (Al-Majali et al. 2009; Shakya et al. 2020).

There was an increase found in bovine brucellosis cases in Azerbaijan during the study period. This may be attributed to a change in the government organization responsible for animal disease control, among other factors. Previously, the surveillance system may have been less robust, possibly due to inadequate resources or inefficiencies in surveillance and prevention measures. However, with the establishment of the Azerbaijan Food Safety Agency (AFSA) to take over veterinary control, there has been a noticeable improvement in the surveillance of diseases such as brucellosis. The involvement of the AFSA likely led to more systematic and rigorous monitoring of animal health, including the detection of brucellosis cases through serological dissemination. On the other hand, we acknowledge the observed discrepancies in brucellosis prevalence data for Azerbaijan between 2018 and 2020. These significant differences, especially in 2018 and in small ruminants, are related to the number of animals tested. In 2018, when the official authority responsible for veterinary control in Azerbaijan transitioned from the Ministry of Agriculture to the newly established Azerbaijan Food Safety Agency (AFSA), limited emphasis was placed on surveillance programs, which resulted in fewer samples being collected during the initial operational phase. In 2018, 19,319 animals were tested, while in the other years over 100,000 animals were tested. This substantial difference in the denominator is due to significant variations in the number of animals tested across these years as the reduced sampling effort directly impacted the prevalence data and the corresponding confidence intervals.

In Armenia, the increase in prevalence over time, particularly in SR, can be attributed to several factors. Firstly, SR graze predominantly on summer pastures, often spread over several regions, sometimes more than 100 kms away from their original communities, increasing the likelihood of contact with other herds and thus, exposure to infection. On the other hand, cattle are often housed in shelters under relatively controlled biosecurity conditions. The fact that SR are only tested once a year may also be of importance. If an animal is not found to be brucellosis positive during the annual test, it will take another year for its infection to be detected. In contrast, cattle are tested every six months, making the identification and removal of brucellosis-positive animals more likely than in SR.

Vaccination of ruminants against brucellosis has been proven to reduce the incidence of the disease in humans in other countries. Nevertheless, even when vaccination campaigns are a key part of control and eradication programs in endemic countries, vaccination on its own is not sufficient for eradication. Brucellosis mass vaccination campaigns must achieve at least 80% coverage to be effective (Zinsstag et al. 2005; Kracalik et al. 2016). However, our study revealed that the average vaccination coverage in the four countries examined was lower than 80%. Low vaccination coverage of SR in Türkiye could be attributed to the inclusion of females and males under six months of age and between 12 and 24 months old, and males over two years old in the census obtained from the Statistical Institute of Türkiye, which is higher than the target SR population of breeding males, female lambs, and kids aged 3–6 months old. On the contrary, cattle vaccination coverage in Türkiye exceeded the average coverage in SR. This is probably due to the use of the female calf population under one year old instead of 3–6 months of age and females under two years old as the denominator in the calculations. The low average cattle vaccination coverage in Azerbaijan and Georgia may be due to the vaccination population used as the denominator in the calculations, which is lower than the actual population due to the lack of fully implemented NAITSs.

According to the results of active surveillance in Armenia and passive surveillance in Türkiye, the prevalence of brucellosis in Armenia (2014–2020) and the incidence in Türkiye (2017–2020) was under 1%. When brucellosis prevalence in animals is less than 1%, a change from vaccination of young animals to a test-and-slaughter strategy should be considered. However, this strategy can be economically challenging as governments must be able to provide enough compensation for farmers (Can 2010; Zhang et al. 2018), and have a NAITS in place to allow for the identification and movement control of animals. There was no NAITS available in Armenia between 2014 and 2020. The absence of compensation practices, as reported in studies in Armenia (Mkrtchyan 2014; Baghiyan and Markosyan 2015), Azerbaijan (Khatibi et al. 2021), and Georgia (World Health Organization 2019), may result in farmers being reluctant to report abortions in livestock, leading to underreporting of health status and mixing positive animals into healthy herds. Alternative, more economical options could be the vaccination of young animals, combined with the elimination of infected animals, which, like the test-and-slaughter strategy, also requires compensation to the farmers. However, herd immunity is established slowly, and there is a need to differentiate vaccinated from infected animals.

The geographical distribution of animal brucellosis can be similar to that of human cases, since high morbidity of human brucellosis has been attributed to a high number of diseased animals (Wolfram et al. 2010; Erbaydar et al. 2012; Aliyev et al. 2022). According to Sargsyan et al. (2019), human brucellosis was unevenly distributed across Armenia between 2006 and 2016, with the majority of cases occurring in rural areas following exposure to livestock, i.e., sheep (71.1%), cattle (69.7%), and goats (42.1%) and the consumption of raw, unpasteurized milk. The endemicity in rural areas found in the current study is consistent with other studies conducted in Mongolia, Georgia, Egypt, and Türkiye (Porphyre et al. 2010; Yumuk and O’Callaghan 2012; Sargsyan et al. 2019). Similar to the findings of Sargsyan et al. (2019), Paronyan et al. (2016) reported that most human brucellosis cases were found in southern Armenia and the rural areas bordering Georgia between 2012 and 2013, with risk factors including the consumption of raw, unpasteurised dairy products and working with animals. The presence of slaughterhouses may also exacerbate the incidence of brucellosis, as illustrated by findings for the Syunik region of Armenia, which had the highest incidence. In our study, we did not have information on the exact location of human brucellosis cases. However, their spatial distribution between 2016 and 2020 was consistent with these findings. On the other hand, the number of human cases in Armenia fluctuated greatly during the study period. This variation in human disease incidence was primarily influenced by the use of passive surveillance, which was based on human reports. In this case, the awareness of people played a crucial role, which was reflected in the results.

In Azerbaijan, the relationship between human and animal brucellosis is less clear: between 1983 and 2009, the incidence of human brucellosis was highest in the west, followed by the eastern part of the country (Kracalik et al. 2016). Nonetheless, our study found the highest SMR of ruminant brucellosis occurred in the central and eastern parts of Azerbaijan. The main factor contributing to this difference is likely to be the prevalence of livestock as the mainstay of the local economy. In these districts, the majority of the population depends on livestock for their livelihoods (Electronic Integrated Disease Surveillance System 2023).

According to the Georgian NCDC, human brucellosis cases are unevenly distributed throughout Georgia. Most cases have been detected in Kakheti and Kvemo Kartli, in the east and southeast, but the actual number of cases throughout the country may be higher due to underreporting (Akhvlediani et al. 2010, 2017; Havas et al. 2012; Sidamonidze et al. 2015). In a study conducted by Akhvlediani et al. in 2010, contact with animals and with animal products were found to be the most common types of exposure among brucellosis patients in Georgia. Similar results were obtained by Havas et al. (2013) and the most significant risk factors for human brucellosis were identified as living in Kakheti and Kvemo-Kartli, working with animals, and being involved in sheep farming or dairy production. Our study also found that between 2016 and 2018 there was a high incidence of human cases in Kakheti and Kvemo-Kartli, as well as the western part of Georgia bordering Türkiye. This could be attributed to the large ruminant population in these regions. In addition, after the National Food Agency of Georgia sampled animals in the Kakheti region, where the livestock system is improved, people in the area may have become interested in their test results and subsequently went to a hospital, which may be why the incidence was found to be higher than in other regions. In contrast, in the capital city of Tbilisi, where no one has cattle, there was no interest in the human population in getting tested. Similar to our findings, the incidence of human brucellosis was higher in the western part of Georgia between 2009 and 2013 compared to 1991–2005, when the eastern region of Georgia recorded almost 3% of the cases (Sidamonidze et al. 2015). According to the results of active surveillance, there have been no brucellosis cases in livestock in the Racha-Lechkumi region and there have been no cases in humans. The prevalence of animal and human brucellosis in the Autonomous Republic of Abkhazia is unknown because of the existing conflicts in the area.

Türkiye has always played an important epidemiological role in the spread of infectious diseases in the region, particularly with its neighbours to the east and southeast, including the Islamic Republic of Iran, Iraq, and Syria (Bora et al. 2016). In both rural and urban areas of Türkiye, brucellosis is a common health problem, according to a number of clinical studies (Aypak et al. 2012). In Türkiye, the high prevalence of ruminant brucellosis in the Eastern, Southeastern, and Central Anatolia regions bordering Armenia, Nakhchivan (a landlocked exclave of Azerbaijan), and the Islamic Republic of Iran, parallels the distribution of human brucellosis cases, and the total number of human cases in these three regions accounts for 85% of the country’s cases (Sümer et al. 2003; Yüce and Alp-Çavuş 2006; Can 2010; Aypak et al. 2012; Erbaydar et al. 2012; Bora et al. 2016). The high seropositivity found in humans in these regions has been attributed to consumption of unpasteurized dairy products and poor socio-economic conditions. The high seropositivity in livestock has been attributed to lax implementation of prevention and control measures in the region, and the uncontrolled movement of animals between Türkiye and its neighbouring countries (Apan et al. 2007; Şahin et al. 2008; Celebi and Atabay 2009; Can 2010; Aypak et al. 2012; Arvas et al. 2013; Bora et al. 2016). Although ruminant brucellosis cases have not been reported in Southeastern Anatolia, this region, along with Eastern Anatolia, experienced the highest incidence in humans. The absence of cases in ruminants in Southeastern Anatolia might suggest a lack of disease reporting.

In addition to underdiagnosed and/or underreported cases in humans (Havas 2011), it is important to determine, at country level, whether the cases reported by national health systems reflect where the patients were infected or where they were diagnosed and treated. Access to healthcare facilities also affects the reporting rates of the disease (Corbel 2006; Havas 2011; Kracalik et al. 2016). The distribution of human cases in the capitals of Armenia, Azerbaijan and Georgia in our study was 5.76%, 14.5%, and 6.20% of all cases between 2016 and 2020, respectively. As suggested by Akhvlediani et al. (2010), it may be that not all patients were sent to hospitals in the capital cities or reported to the national healthcare systems. Therefore, the human cases reported in the current study may not reflect the accurate incidence.

A limitation of this study is that the animal data collected depended on the available and shared national data. The calculation of vaccination coverage also differed between Azerbaijan, Georgia and Türkiye: in Azerbaijan and Georgia, the denominator used in the calculations were based on the estimated proportion of the total population expected to be vaccinated against brucellosis. However, in Türkiye the denominator for SR was the number of females and males under six months of age and between 12 and 24 months old, as the exact proportion of the females and males is unknown, and males over two years of age. For cattle in Türkiye, the denominator was the female calf population under one year old instead of 3–6 months of age, and under two years of age, as females were revaccinated 4–12 months after their first vaccination. In addition, data on human incidence in Türkiye were only open-access available for 2017 and did not allow us to analyze the status in the country for other years.

## Conclusion

Brucellosis has a significant impact on animal and public health in many countries around the world. It is endemic in the four countries included in this study, where the disease was found to be unevenly distributed, with higher incidences in areas bordering neighbouring countries, likely due to transboundary movements of animals and grazing in common pastures. Although the prevalence in animals was low, it showed a slight increase during the study period. The incidence in humans varied slightly between the regions of the countries concerned. There was a mismatch found between the incidence of brucellosis in animals and humans, despite the expectation that they would follow similar spatial–temporal patterns. Underreporting of cases can be regarded as a significant issue in both humans and livestock and is influenced by a series of socioeconomic factors.

For the successful control and eventual eradication of brucellosis, it is important to reinforce veterinary services and fully adopt a NAITS that can provide accurate and timely information for the effective planning, implementation, and monitoring of surveillance, control (e.g., test-and-slaughter) and vaccination programs, as well as for tracing animal movements. A sound compensation mechanism is also key to encourage reporting and the collaboration of farmers. In addition, regional cooperation is also critical for the strict control of irregular animal movements between neighbouring countries. The control/eradication strategy of choice needs to carefully consider the above constraints and be sustainable over time, in terms of funds, personnel and other resources. Surveillance activities should be conducted continuously to contribute to a better understanding of the epidemiological situation, thus allowing the improvement and adaptation of national programs towards the prevention and eradication of brucellosis. Finally, the successful implementation of a national brucellosis control program also requires close collaboration between public health and veterinary services to share data and coordinate control plans and field activities. Other relevant stakeholders, including actors along the meat and milk value chains, as well as consumers, need to be part of the effort, particularly when it comes to raising public awareness of the risk factors for brucellosis transmission routes.

## Supplementary Information

Below is the link to the electronic supplementary material.

Supplementary file1 (DOCX 45 KB)

## Data Availability

The data sets generated and/or analyzed during the current study are available from the corresponding author upon reasonable request.
