# Supplementary material for: Ruminant and human brucellosis situation in Türkiye and the Caucasus
Source: Trop Anim Health Prod. 2025 Jul 11;57(6):296. doi: 10.1007/s11250-025-04525-1 (PMC12254074; doi:10.1007/s11250-025-04525-1)
Supplement: Supplementary file 1 — Supplementary file1 (DOCX 45 KB) [file 11250_2025_4525_MOESM1_ESM.docx]

Ruminant and human brucellosis situation in Türkiye and the Caucasus

Tropical Animal Health and Production

Ipek Keskin Fernandez-Georges, Sheina Macy Manalo, Margarida Arede, Giovanna Ciaravino, Daniel Beltrán-Alcrudo, Jordi Casal, Eran Raizman, Jeyhun Aliyev, Tengiz Chaligava, Tigran Markosyan, Alberto Allepuz

Department of Animal Health and Anatomy, Faculty of Veterinary Medicine, Autonomous University of Barcelona (UAB), Barcelona-Spain, alberto.allepuz@uab.cat

**Table 1** Brucellosis incidence in humans in Armenia by marz (2016-2020)

| Year | 2016 | | 2017 | | 2018 | | 2019 | | 2020 | |
| --- | --- | --- | --- | --- | --- | --- | --- | --- | --- | --- |
| Marz | Incidence* | Cases / Population | Incidence* | Cases / Population | Incidence* | Cases / Population | Incidence* | Cases / Population | Incidence* | Cases / Population |
| Aragatsotn | 36.6 | 47/128,300 | 37.1 | 47/126,700 | 17.7 | 22/124,400 | 34.5 | 43/124,700 | 17.6 | 22/124,700 |
| Ararat | 12.4 | 32/258,000 | 19.8 | 51/257,700 | 13.3 | 34/256,200 | 11.7 | 30/257,200 | 5.05 | 13/257,400 |
| Armavir | 7.53 | 20/265,600 | 12.1 | 32/264,500 | 8.73 | 23/263,400 | 9.07 | 24/264,700 | 5.29 | 14/264,800 |
| Gegharkunik | 9.14 | 21/229,700 | 10.0 | 23/229,000 | 7.92 | 18/227,200 | 8.80 | 20/227,400 | 14.1 | 32/227,300 |
| Kotayk | 13.9 | 35/251,900 | 26.7 | 67/251,400 | 10.0 | 25/251,200 | 5.56 | 14/251,700 | 1.59 | 4/252,100 |
| Lori | 1.37 | 3/219,300 | 2.78 | 6/215,800 | 4.22 | 9/213,500 | 5.16 | 11/213,100 | 0.47 | 1/212,200 |
| Shirak | 13.9 | 33/237,400 | 20.1 | 47/234,100 | 7.37 | 17/230,800 | 18.1 | 42/231,500 | 1.30 | 3/230,300 |
| Syunik | 49.7 | 69/138,800 | 41.9 | 58/138,400 | 18.2 | 25/137,400 | 28.6 | 39/136,400 | 24.5 | 33/134,600 |
| Tavush | 0.00 | 0/123,900 | 0.81 | 1/122,900 | 0.00 | 0/121,100 | 0.00 | 0/120,800 | 0.00 | 0/119,700 |
| Vayots Dzor | 9.98 | 5/50,100 | 18.3 | 9/49,300 | 18.5 | 9/48,700 | 24.8 | 12/48,400 | 4.20 | 2/47,600 |
| Yerevan | 1.02 | 11/1,076,600 | 1.95 | 21/1,079,400 | 1.75 | 19/1,083,600 | 1.01 | 11/1,092,000 | 0.73 | 8/1,096,100 |

*Incidences were calculated in cases per 100,000 population.

**Table 2** Brucellosis incidence in humans in the districts of Azerbaijan (2016-2020)

| Year | 2016 | | 2017 | | 2018 | | 2019 | | 2020 | |
| --- | --- | --- | --- | --- | --- | --- | --- | --- | --- | --- |
| District | Incidence* | Cases / Population | Incidence* | Cases / Population | Incidence* | Cases / Population | Incidence* | Cases / Population | Incidence* | Cases / Population |
| Absheron | 7.23 | 15/207,500 | 5.24 | 11/210,000 | 7.53 | 16/212,600 | 1.40 | 6/428,500 | 0.70 | 3/429,600 |
| Agdam | 1.01 | 2/197,700 | 0.50 | 1/200,000 | 0.00 | 0/202,200 | 1.71 | 3/175,200 | 0.00 | 0/176,400 |
| Agdash | 0.93 | 1/107,900 | 0.92 | 1/108,700 | 3.64 | 4/109,900 | 1.92 | 2/104,000 | 0.95 | 1/104,900 |
| Agjabadi | 12.0 | 16/133,300 | 5.20 | 7/134,500 | 7.38 | 10/135,500 | 6.79 | 9/132,600 | 3.75 | 5/133,200 |
| Agstafa | 2.31 | 2/86,600 | 4.59 | 4/87,200 | 1.14 | 1/87,900 | 0.00 | 0/84,800 | 0.00 | 0/85,100 |
| Agsu | 3.83 | 3/78,300 | 2.53 | 2/79,200 | 9.99 | 8/80,100 | 7.67 | 6/78,200 | 5.08 | 4/78,800 |
| Astara | 0.00 | 0/106,500 | 0.00 | 0/107,600 | 2.76 | 3/108,600 | 0.00 | 0/108,300 | 0.00 | 0/109,100 |
| Babek | 0.00 | 0 /74,300 | 0.00 | 0/75,000 | 0.00 | 0/75,600 | 0.00 | 0/76,200 | 0.00 | 0/76,500 |
| Baku city | 2.49 | 56/2,245,800 | 3.98 | 90/2,262,600 | 2.20 | 50/2,277,500 | 2.31 | 54/2,333,100 | 1.33 | 31/2,334,400 |
| Balakan | 2.07 | 2/96,800 | 0.00 | 0/97,600 | 2.03 | 2/98,300 | 0.00 | 0/97,900 | 0.00 | 0/98,800 |
| Barda | 3.24 | 5/154,200 | 12.2 | 19/155,400 | 4.48 | 7/156,300 | 8.47 | 13/153,400 | 0.00 | 0/154,100 |
| Beylagan | 10.4 | 10/96,400 | 7.18 | 7/97,500 | 26.4 | 26/98,600 | 20.3 | 20/98,300 | 3.03 | 3/99,100 |
| Bilasuvar | 0.99 | 1/100,900 | 1.95 | 2/102,400 | 0.00 | 0/103,800 | 0.98 | 1/102,300 | 0.97 | 1/103,400 |
| Dashkasan | 14.4 | 5/34,800 | 11.4 | 4/35,000 | 17.1 | 6/35,100 | 12.2 | 4/32,900 | 3.03 | 1/33,000 |
| Fizuli | 2.32 | 3/129,400 | 5.35 | 7/130,900 | 3.78 | 5/132,400 | 1.56 | 2/127,800 | 1.56 | 2/128,600 |
| Gadabay | 3.02 | 3/99,200 | 10.0 | 10/99,800 | 3.98 | 4/100,400 | 33.3 | 32/96,000 | 12.3 | 12/97,700 |
| Ganja city | 5.73 | 19/331,400 | 1.20 | 4/332,600 | 3.29 | 11/334,000 | 4.17 | 14/336,125 | 0.29 | 1/339,184 |
| Goranboy | 5.86 | 6/102,400 | 2.90 | 3/103,300 | 6.72 | 7/104,100 | 9.34 | 9/96,400 | 0.00 | 0/98,100 |
| Goychay | 2.54 | 3/118,300 | 2.51 | 3/119,400 | 1.66 | 2/120,600 | 5.19 | 6/115,700 | 3.43 | 4/116,500 |
| Goygol | 7.96 | 5/62,800 | 17.4 | 11/63,400 | 9.39 | 6/63,900 | 4.74 | 3/63,300 | 0.00 | 0/63,700 |
| Hajigabul | 4.08 | 3/73,600 | 4.02 | 3/74,700 | 2.65 | 2/75,600 | 0.00 | 0/73,600 | 2.70 | 2/74,100 |
| Imishli | 15.7 | 20/127,100 | 17.1 | 22/128,500 | 22.3 | 29/129,800 | 9.38 | 12/128,000 | 3.10 | 4/128,900 |
| Ismailli | 5.85 | 5/85,500 | 3.48 | 3/86,100 | 3.46 | 3/86,700 | 3.52 | 3/85,300 | 1.17 | 1/85,700 |
| Jabrayil | 3.80 | 3/79,000 | 6.26 | 5/79,900 | 11.1 | 9/80,800 | 11.2 | 8/71,400 | 6.94 | 5/72,000 |
| Jalilabad | 3.69 | 8/216,600 | 3.64 | 8/219,500 | 1.80 | 4/222,400 | 2.41 | 5/207,800 | 1.90 | 4/210,100 |
| Julfa | 0.00 | 0/46,100 | 0.00 | 0/46,400 | 0.00 | 0/46,700 | 0.00 | 0/47,000 | 0.00 | 0/47,300 |
| Kalbajar | 0.00 | 0/91,100 | 0.00 | 0/92,100 | 0.00 | 0/93,100 | 0.00 | 0/71,300 | 1.39 | 1/71,900 |
| Kengerli | 0.00 | 0/31,800 | 0.00 | 0/32,200 | 0.00 | 0/32,400 | 0.00 | 0/32,800 | 0.00 | 0/32,900 |
| Khachmaz | 4.58 | 8/174,800 | 0.57 | 1/176,300 | 2.81 | 5/178,100 | 1.18 | 2/169,700 | 1.75 | 3/171,100 |
| Khankendy | 0.00 | 0/55,700 | 0.00 | 0/55,800 | 0.00 | 0/55,800 | 0.00 | 0/4,300 | 0.00 | 0/4,300 |
| Khizi | 24.1 | 4/16,600 | 17.9 | 3/16,800 | 53.3 | 9/16,900 | 12.3 | 2/16,300 | 12.2 | 2/16,400 |
| Khojali | 0.00 | 0/28,000 | 0.00 | 0/28,300 | 0.00 | 0/28,600 | 0.00 | 0/11,100 | 0.00 | 0/11,200 |
| Khojavend | 0.00 | 0/43,400 | 0.00 | 0/43,600 | 0.00 | 0/43,800 | 0.00 | 0/9,400 | 0.00 | 0/10,400 |
| Kurdamir | 12.2 | 14/114,400 | 13.8 | 16/115,600 | 38.6 | 45/116,700 | 55.7 | 64/115,000 | 7.77 | 9/115,900 |
| Lachin | 0.00 | 0/76,100 | 0.00 | 0/76,900 | 1.29 | 1/77,700 | 4.09 | 3/73,300 | 0.00 | 0/73,800 |
| Lankaran | 0.89 | 2/225,200 | 0.44 | 1/226,900 | 0.87 | 2/228,700 | 0.89 | 2/224,100 | 1.33 | 3/225,000 |
| Lerik | 0.00 | 0/82,800 | 1.19 | 1/83,800 | 3.54 | 3/84,700 | 4.84 | 4/82,700 | 1.20 | 1/83,600 |
| Masally | 4.06 | 9/221,500 | 2.23 | 5/223,800 | 1.33 | 3/225,800 | 5.87 | 13/221,600 | 0.45 | 1/222,900 |
| Mingachevir | 4.84 | 5/103,200 | 1.91 | 2/104,500 | 0.00 | 0/105,400 | 0.99 | 1/101,400 | 0.00 | 0/101,700 |
| Naftalan | 0.00 | 0/10,000 | 0.00 | 0/10,100 | 0.00 | 0/10,200 | 0.00 | 0/8,500 | 0.00 | 0/8,600 |
| Nakhichevan | 0.00 | 0/92,100 | 0.00 | 0/92,900 | 0.00 | 0/93,700 | 0.00 | 0/94,600 | 0.00 | 0/95,100 |
| Neftchala | 2.31 | 2/86,500 | 2.29 | 2/87,300 | 5.68 | 5/88,000 | 3.51 | 3/85,400 | 1.17 | 1/85,800 |
| Oguz | 2.30 | 1/43,500 | 22.7 | 10/44,000 | 6.77 | 3/44,300 | 0.00 | 0/43,300 | 2.29 | 1/43,700 |
| Ordubad | 0.00 | 0/49,500 | 0.00 | 0/49,700 | 0.00 | 0/50,000 | 0.00 | 0/50,200 | 0.00 | 0/50,400 |
| Qabala | 10.5 | 11/104,400 | 10.4 | 11/105,500 | 7.50 | 8/106,600 | 9.47 | 10/105,600 | 4.69 | 5/106,500 |
| Qakh | 3.57 | 2/56,100 | 10.6 | 6/56,500 | 19.3 | 11/56,900 | 20.9 | 12/57,400 | 0.00 | 0/57,600 |
| Qazakh | 7.31 | 7/95,800 | 11.4 | 11/96,700 | 12.3 | 12/97,600 | 5.29 | 5/94,500 | 0.00 | 0/95,000 |
| Qobustan | 19.8 | 9/45,400 | 10.8 | 5/46,100 | 8.57 | 4/46,700 | 0.00 | 0/44,700 | 0.00 | 0/45,300 |
| Quba | 2.38 | 4/168,400 | 6.47 | 11/170,000 | 9.32 | 16/171,700 | 3.03 | 5/165,200 | 1.80 | 3/166,500 |
| Qubadli | 0.00 | 0/40,200 | 0.00 | 0/40,700 | 0.00 | 0/41,100 | 0.00 | 0/36,200 | 0.00 | 0/36,500 |
| Qusar | 0.00 | 0/96,200 | 0.00 | 0/97,200 | 0.00 | 0/98,100 | 4.01 | 4/99,800 | 1.00 | 1/100,400 |
| Saatly | 29.5 | 31/105,100 | 10.3 | 11/106,500 | 5.57 | 6/107,800 | 2.81 | 3/106,900 | 2.79 | 3/107,700 |
| Sabirabad | 6.37 | 11/172,700 | 6.29 | 11/174,800 | 4.53 | 8/176,700 | 4.03 | 7/173,800 | 0.57 | 1/175,300 |
| Sadarak | 0.00 | 0/15,800 | 0.00 | 0/15,900 | 0.00 | 0/15,900 | 0.00 | 0/22,600 | 0.00 | 0/22,700 |
| Salyan | 0.00 | 0/135,600 | 1.46 | 2/137,100 | 3.61 | 5/138,600 | 2.95 | 4/135,800 | 0.00 | 0/136,700 |
| Samukh | 22.8 | 13/57,100 | 8.67 | 5/57,700 | 13.7 | 8/58,300 | 12.2 | 7/57,200 | 1.74 | 1 /57,500 |
| Shabran | 13.8 | 8/58,000 | 1.70 | 1 /58,700 | 3.37 | 2/59,300 | 3.51 | 2/56,900 | 0.00 | 0/57,300 |
| Shakhbuz | 0.00 | 0/24,900 | 0.00 | 0/25,100 | 0.00 | 0/25,200 | 0.00 | 0/25,300 | 0.00 | 0/25,300 |
| Shaki | 2.17 | 4/184,200 | 2.70 | 5/185,400 | 1.61 | 3/186,600 | 0.55 | 1/180,900 | 0.00 | 0/181,900 |
| Shamakhi | 4.87 | 5/102,700 | 10.6 | 11/103,900 | 12.4 | 13/105,100 | 4.90 | 5/102,100 | 0.00 | 0/103,100 |
| Shamkir | 5.64 | 12/212,700 | 14.4 | 31/215,000 | 18.0 | 39/217,200 | 23.1 | 49/212,200 | 10.3 | 22/213,400 |
| Sharur | 0.00 | 0/114,600 | 0.00 | 0/115,600 | 0.00 | 0/116,600 | 0.00 | 0/111,100 | 0.00 | 0/111,500 |
| Shirvan | 0.00 | 0/85,000 | 2.33 | 2/85,800 | 3.46 | 3/86,600 | 0.00 | 0/83,700 | 0.00 | 0/84,300 |
| Shusha | 0.00 | 0/33,400 | 0.00 | 0/33,800 | 0.00 | 0/34,200 | 0.00 | 0/22,300 | 0.00 | 0/24,600 |
| Siazan | 2.42 | 1/41,400 | 0.00 | 0/41,900 | 9.48 | 4/42,200 | 2.46 | 1/40,700 | 2.44 | 1/41,000 |
| Sumqayit city | 2.65 | 9/339,000 | 11.7 | 40/341,200 | 4.37 | 15/343,100 | 3.07 | 13/423,100 | 0.71 | 3/424,200 |
| Tartar | 4.84 | 5/103,200 | 2.90 | 3/103,600 | 0.96 | 1/104,200 | 3.79 | 3/79,200 | 5.03 | 4/79,500 |
| Tovuz | 8.12 | 14/172,400 | 3.45 | 6/174,000 | 8.55 | 15/175,500 | 1.16 | 2/172,400 | 0.00 | 0/43,700 |
| Ujar | 2.31 | 2/86,600 | 1.14 | 1 /87,700 | 0.00 | 0/88,600 | 2.42 | 2/82,800 | 0.00 | 0/83,400 |
| Yardymli | 3.07 | 2/65,200 | 1.51 | 1/66,100 | 2.99 | 2/67,000 | 0.00 | 0/65,700 | 0.00 | 0/66,500 |
| Yevlakh | 6.31 | 8/126,700 | 3.92 | 5/127,400 | 3.89 | 5/128,500 | 6.35 | 8/125,900 | 1.58 | 2/126,900 |
| Zangilan | 0.00 | 0/44,000 | 0.00 | 0/44,400 | 0.00 | 0/44,800 | 0.00 | 0/42,100 | 0.00 | 0/42,300 |
| Zaqatala | 1.58 | 2/126,900 | 3.91 | 5/127,800 | 0.00 | 0/128,700 | 3.13 | 4/127,700 | 0.78 | 1/128,400 |
| Zardab | 3.47 | 2/57,700 | 0.00 | 0/58,200 | 6.80 | 4/58,800 | 6.90 | 4/58,000 | 0.00 | 0/58,300 |

*Incidences were calculated in cases per 100,000 population.

**Table 3** Brucellosis incidence in humans in Georgia by region (2016-2020)

| Year | 2016 | | 2017 | | 2018 | | 2019 | | 2020 | |
| --- | --- | --- | --- | --- | --- | --- | --- | --- | --- | --- |
| Region | Incidence* | Cases / Population | Incidence* | Cases / Population | Incidence* | Cases / Population | Incidence* | Cases / Population | Incidence* | Cases / Population |
| Abkhazia | 0.82 | 2/243,564 | 0.00 | 0/243,936 | 0.00 | 0/244,832 | 0.41 | 1/245,246 | 0.00 | 0/245,424 |
| Adjara | 2.35 | 8/340,186 | 0.87 | 3/343,017 | 0.58 | 2/346,347 | 1.15 | 4/349,028 | 0.28 | 1/351,892 |
| Guria | 2.67 | 3/112,432 | 2.69 | 3/111,457 | 1.81 | 2/110,524 | 3.66 | 4/109,396 | 1.85 | 2/108,099 |
| Imereti | 2.86 | 15/523,667 | 1.75 | 9/514,401 | 1.58 | 8/507,038 | 4.02 | 20/497,396 | 2.05 | 10/486,983 |
| Kakheti | 25.8 | 82/317,776 | 31.0 | 98/315,883 | 22.9 | 72/314,681 | 23.4 | 73/312,453 | 17.7 | 55/310,051 |
| Kvemo Kartli | 14.0 | 60/428,044 | 12.3 | 53/429,669 | 12.5 | 54/432,264 | 9.00 | 39/433,162 | 7.37 | 32/434,241 |
| Mtskheta-Mtianeti | 8.50 | 8/94,079 | 7.45 | 7/93,903 | 5.32 | 5/93,897 | 15.0 | 14/93,636 | 6.43 | 6/93,343 |
| Samegrelo-Zemo Svaneti | 1.22 | 4/328,371 | 3.08 | 10/324,168 | 2.81 | 9/320,805 | 2.21 | 7/316,195 | 2.25 | 7/311,113 |
| Samtskhe-Javakheti | 10.7 | 17/158,735 | 10.2 | 16/157,166 | 5.77 | 9/155,903 | 2.60 | 4/154,139 | 1.31 | 2/152,114 |
| Shida Kartli | 0.38 | 1/261,879 | 1.92 | 5/260,434 | 2.31 | 6/259,251 | 2.33 | 6/257,279 | 1.57 | 4/255,124 |
| Tbilisi | 1.24 | 14/1,131,962 | 1.05 | 12/1,145,474 | 0.69 | 8/1,158,677 | 1.37 | 16/1,171,079 | 0.59 | 7/1,184,818 |
| Racha-Lechkhumi and Kvemo Svaneti | 0.00 | 0/31,505 | 0.00 | 0/30,802 | 0.00 | 0/30,246 | 0.00 | 0/29,701 | 0.00 | 0/29,080 |

*Incidences were calculated in cases per 100,000 population.

**Table 4** Brucellosis incidence in humans in the provinces of Türkiye (2017)

| Province | Incidence | Province | Incidence | Province | Incidence | Province | Incidence |
| --- | --- | --- | --- | --- | --- | --- | --- |
| Adana | 5.2 | Bursa | 1.9 | Izmir | 2.7 | Nevsehir | 2.7 |
| Adiyaman | 18.7 | Canakkale | 14.5 | Kahramanmaras | 10.3 | Nigde | 17.0 |
| Afyon | 11.6 | Cankiri | 5.9 | Karabuk | 1.6 | Ordu | 3.6 |
| Agri | 19.0 | Corum | 11.2 | Karaman | 16.2 | Osmaniye | 12.5 |
| Aksaray | 25.1 | Denizli | 3.3 | Kars | 82.4 | Rize | 0.6 |
| Amasya | 2.4 | Diyarbakir | 12.1 | Kastamonu | 0.5 | Sakarya | 0.8 |
| Ankara | 1.9 | Duzce | 1.9 | Kayseri | 7.6 | Samsun | 1.0 |
| Antalya | 3.8 | Edirne | 8.4 | Kilis | 16.9 | Sanliurfa | 15.9 |
| Ardahan | 24.7 | Elazig | 8.7 | Kirikkale | 2.5 | Siirt | 45.0 |
| Artvin | 1.2 | Erzincan | 14.7 | Kirklareli | 3.1 | Sinop | 0.0 |
| Aydin | 1.7 | Erzurum | 19.5 | Kirsehir | 17.9 | Sirnak | 37.6 |
| Balikesir | 8.3 | Eskisehir | 11.5 | Kocaeli | 0.8 | Sivas | 8.9 |
| Bartin | 2.1 | Gaziantep | 4.5 | Konya | 12.9 | Tekirdag | 3.3 |
| Batman | 60.0 | Giresun | 4.6 | Kutahya | 6.1 | Tokat | 9.6 |
| Bayburt | 14.9 | Gumushane | 5.3 | Malatya | 9.4 | Trabzon | 4.5 |
| Bilecik | 1.8 | Hakkari | 62.4 | Manisa | 8.6 | Tunceli | 6.1 |
| Bingol | 15.7 | Hatay | 11.7 | Mardin | 9.5 | Usak | 7.1 |
| Bitlis | 61.2 | Igdir | 76.0 | Mersin | 3.0 | Van | 57.6 |
| Bolu | 7.3 | Isparta | 24.2 | Mugla | 0.9 | Yalova | 0.4 |
| Burdur | 9.8 | Istanbul | 1.1 | Mus | 7.2 | Yozgat | 19.6 |
| Zonguldak | 0.7 |  |  |  |  |  |  |
